# Evaluation of therapeutic compliance in psychiatry

**DOI:** 10.1192/j.eurpsy.2023.1842

**Published:** 2023-07-19

**Authors:** H. Abrebak, F. Z. Chamsi, A. Essafi, S. Radi, A. Taqui, A. El Ammouri

**Affiliations:** 1 Faculty of medicine of tangier; 2 Faculté de médecine de tanger, Tangier, Morocco

## Abstract

**Introduction:**

Therapeutic non-adherence is a problem frequently encountered in patients suffering from psychotic disorders. It has consequences on the quality of life and on the prognosis of the evolution of the disease. It is essential to understand the causes in order to best support the patient towards adherence to care.

**Objectives:**

The objective of the study is to evaluate the therapeutic observance of patients hospitalized in the women’s department of the psychiatric hospital and to collect the reasons for therapeutic non-observance, as well as to identify the desired themes for the implementation group therapeutic education workshops.

**Methods:**

It is a monocentric and prospective study, carried out in the women’s department through individual interviews on day 7 +/- 2 of the admission of patients to the psychiatric hospital of Tangier. The inclusion criteria are:

- the patient’s consent,
- the autonomous taking of a treatment before admission,
- sufficient communication skills

The psychometric tool used during this study is the MARS scale (Medication Adherence Report Scale) which allows the patient to assess his compliance, by answering 10 questions, assigning himself a score between 0 and 10 with a good compliance from 8/10. The discussion following the questionnaire makes it possible to identify the reason(s) for non-compliance and the themes to be addressed to improve compliance.

**Results:**

Our first initial results were calculated from 60 patients admitted to hospital. Among them, 35 met the criteria for inclusion in the study. The interview takes place within an average of 8.0 ± 2.3 days. The average age is 40 ± 15 years. Patients present with schizophrenia in 80% of cases, bipolarity (8%) or borderline personality disorder (3%). Nine percent of patients have no diagnosis. Patients take an average of 2.5 drugs [1; 5] before hospitalization. The average MARS score is 5.6 ± 2.6. The reasons for non-adherence identified by patients are:

- The presence of side effects,
- Lack of means
- Feeling of healing
- The weariness of a long treatment
- Inefficiency,
- fear of interactions in case of toxic consumption
- five patients declared observing and did not identify any reason for non-compliance

**Conclusions:**

Our study has made it possible to better understand the difficulties and support needs of patients to improve their adherence to care. As a follow-up to this work, a multidisciplinary discussion will allow the setting up of group therapeutic education workshops around the identified themes.

**Disclosure of Interest:**

None Declared

